# Expression Plasmids for Use in *Candida glabrata*

**DOI:** 10.1534/g3.113.006908

**Published:** 2013-10-01

**Authors:** Rebecca E. Zordan, Yuxia Ren, Shih-Jung Pan, Giuseppe Rotondo, Alejandro De Las Peñas, Joseph Iluore, Brendan P. Cormack

**Affiliations:** * Department of Molecular Biology and Genetics, Johns Hopkins University School of Medicine, Baltimore, Maryland 21205

**Keywords:** *Candida glabrata*, expression vector, macrophage, inducible, *MET*3

## Abstract

We describe a series of CEN/ARS episomal plasmids containing different *Candida glabrata* promoters, allowing for a range of constitutive or regulated expression of proteins in *C. glabrata*. The set of promoters includes three constitutive promoters (*EGD2*pr, *HHT2*pr, *PDC1*pr), two macrophage/phagocytosis-induced promoters (*ACO2*pr, *LYS21*pr), and one nutritionally regulated promoter (*MET3*pr). Each promoter was cloned into two plasmid backbones that differ in their selectable marker, *URA3*, or the dominant-selectable *NAT1* gene, which confers resistance to the drug nourseothricin. Expression from the 12 resulting plasmids was assessed using GFP as a reporter and flow cytometry or quantitative reverse-transcription polymerase chain reaction to assess expression levels. Together this set of plasmids expands the toolkit of expression vectors available for use with *C. glabrata*.

*Candida glabrata* is an important fungal pathogen, causing both superficial and deep infections. *C. glabrata* has several traits that have been linked to virulence, including a repertoire of adhesins, the ability to adapt to and modify the macrophage phagolysosomal environment, and an inherent resistance to azole antifungals (Pfaller 2012). The organism is genetically tractable, and both transposon mutagenesis and reverse genetic approaches have been used to generate mutants (Petter and Kwon-Chung 1996; Castano *et al.* 2003; Kaur *et al.* 2004). To expand the range of tools available for *C. glabrata*, we made a panel of expression vectors. The goal was to create a series of *Escherichia coli–C. glabrata* shuttle vectors containing different *C. glabrata* promoters and a choice of yeast-selectable markers. Analogous shuttle vectors have been designed for use in *Saccharomyces cerevisiae* and other yeasts (Sikorski and Hieter 1989; Christianson *et al.* 1992; Gould *et al.* 1992; Mumberg *et al.* 1995; Chen 1996; Brachmann *et al.* 1998; Adams *et al.* 2005; Chee and Haase 2012).

This series of cloning vectors contains a pMB1 (ColE1 family) origin of replication and Ap^R^ marker for propagation in *E. coli*. A *C. glabrata* CEN sequence and an ARS element permit propagation and stable maintenance of the plasmid. A polylinker positioned between the *C. glabrata*–specific promoter and a transcriptional terminator facilitate cloning of genes of interest under control of a given promoter. We constructed this series of plasmids with a range of *C. glabrata* promoters; there are three constitutively active promoters, two macrophage-induced promoters, and one nutritionally regulated promoter. These options allow researchers to vary the level of expression for a target gene, and because the plasmids use the same polylinker, target genes can be easily shuttled between backbones with different promoters. Additionally, there is a choice of two selectable markers for use in *C. glabrata*, the *URA3* auxotrophic marker or the dominant NAT drug-resistance cassette.

We describe the construction of these plasmids and quantify the expression level driven by each promoter by monitoring GFP expression by flow cytometry or by quantitative reverse-transcription polymerase chain reaction (qRT-PCR). This set of vectors will facilitate regulated or constitutive expression of genes in *C. glabrata* and expands the genetic toolbox available for *C. glabrata*.

## Materials and Methods

### Growth media

*C. glabrata* was routinely grown on YPD media (10 g/liter yeast extract, 20 g/liter peptone, 2% dextrose) at 30°C. All solid media contained 2% agar. Nourseothricin (NAT; clonNAT; Werner BioAgents) was supplemented to liquid YPD media at 50 μg/ml and to solid YPD media at 100 μg/ml to select for *C. glabrata* strains containing pCN vectors. Strains containing *URA3*-marked plasmids (pCU series) were grown in SD-Ura (1.7 g/liter yeast nitrogen base without amino acids or ammonium sulfate, 5 g/liter ammonium sulfate, 6 g/liter casamino acids, 2% dextrose).

The *MET3* promoter is controlled by the presence of methionine and cysteine in the media. Media lacking methionine, cysteine, and uracil was used to induce expression, whereas addition of Met and Cys (2 mM each) was used to repress the *MET3* promoter. SD−Met−Cys−Ura and SD+Met+Cys−Ura media were made using +Met+Cys−Ura or −Met−Cys−Ura amino acid mixtures, respectively. For the pCN-MET3 vectors, SED media (1.7 g/liter yeast nitrogen base without amino acids or ammonium sulfate, 1 g/liter monosodium glutamate, 2% dextrose) is used instead of standard SD, because NAT is not inhibitory in the presence of ammonium sulfate (Cheng *et al.* 2000). The resulting SED−Met−Cys−Ura and SED+Met+Cys−Ura media were supplemented with Nat (50 μg/ml in liquid, 100 μg/ml in plates). Supporting Information, Table S1 shows the components of each amino acid mixture.

Experiments testing plasmid maintenance required counterselection against the *URA3* marker using 5-fluoroorotic acid (5-FOA). For these purposes, colonies were grown on 5-FOA plates (1.7 g/liter yeast nitrogen base without amino acids or ammonium sulfate, 5 g/liter ammonium sulfate, 6 g/liter casamino acids, 25 mg/liter uracil, 1 g/liter 5-FOA, 2% dextrose, 2% agar).

### Strains and transformation

*E. coli* was grown in LB and transformants were selected in LB plus 100 μg/ml carbenicillin. All *C. glabrata* strains used in this study are listed in Table 1 (Cormack and Falkow 1999). *C. glabrata* strains were transformed using a modified LiOAc transformation protocol as described previously (Castano *et al.* 2003). pCU plasmids were transformed into strain BG14 and transformants selected on SD−Ura plates. pCN plasmids were transformed into strain BG2 and transformants were selected in the presence of NAT (100 μg/ml).

**Table 1:** *C. glabrata* strains used in this study

Strain	Description	Genotype	Parent Strain	Source
General use				
BG2	Clinical isolate	Wild-type clinical isolate	NA	Cormack and Falkow 1999
BG14	Ura− version of BG2	*ura3*Δ::Tn903 G418^R^	BG2	Cormack and Falkow 1999
CBS138	Clinical isolate	Wild-type clinical isolate (ATCC#2001)	NA	ATCC
BG2389	qPCR control	*ura3*Δ::Tn903 G418^R^	BG14	S. Pan, unpublished
		*sla2*Δ::Hyg^R^ (*URA3*, Amp^R^)		
Constitutive promoters (*URA3*-marked)				
BG3316	pCU-EGD2 #1	*ura3*Δ::Tn903 G418^R^, pCU-EGD2	BG14	This work
BG3317	pCU-EGD2 #2	*ura3*Δ::Tn903 G418^R^, pCU-EGD2	BG14	This work
BG2988	pCU-EGD2-GFP #1	*ura3*Δ::Tn903 G418^R^, pCU-EGD2-GFP	BG14	This work
BG3147	pCU-EGD2-GFP #2	*ura3*Δ::Tn903 G418^R^, pCU-EGD2-GFP	BG14	This work
BG3318	pCU-HHT2 #1	*ura3*Δ::Tn903 G418^R^, pCU-HHT2	BG14	This work
BG3319	pCU-HHT2 #2	*ura3*Δ::Tn903 G418^R^, pCU-HHT2	BG14	This work
BG2989	pCU-HHT2-GFP #1	*ura3*Δ::Tn903 G418^R^, pCU-HHT2-GFP	BG14	This work
BG3148	pCU-HHT2-GFP #2	*ura3*Δ::Tn903 G418^R^, pCU-HHT2-GFP	BG14	This work
BG3320	pCU-PDC1 #1	*ura3*Δ::Tn903 G418^R^, pCU-PDC1	BG14	This work
BG3321	pCU-PDC1 #2	*ura3*Δ::Tn903 G418^R^, pCU-PDC1	BG14	This work
BG2990	pCU-PDC1-GFP #1	*ura3*Δ::Tn903 G418^R^, pCU-PDC1-GFP	BG14	This work
BG3149	pCU-PDC1-GFP #2	*ura3*Δ::Tn903 G418^R^, pCU-PDC1-GFP	BG14	This work
Constitutive promoters (NAT^R^-marked)				
BG3328	pCN-EGD2 #1	pCN-EGD2	BG2	This work
BG3329	pCN-EGD2 #2	pCN-EGD2	BG2	This work
BG3342	pCN-EGD2-GFP #1	pCN-EGD2-GFP	BG2	This work
BG3343	pCN-EGD2-GFP #2	pCN-EGD2-GFP	BG2	This work
BG3330	pCN-HHT2 #1	pCN-HHT2	BG2	This work
BG3331	pCN-HHT2 #2	pCN-HHT2	BG2	This work
BG3344	pCN-HHT2-GFP #1	pCN-HHT2-GFP	BG2	This work
BG3345	pCN-HHT2-GFP #2	pCN-HHT2-GFP	BG2	This work
BG3332	pCN-PDC1 #1	pCN-PDC1	BG2	This work
BG3333	pCN-PDC1 #2	pCN-PDC1	BG2	This work
BG3346	pCN-PDC1-GFP #1	pCN-PDC1-GFP	BG2	This work
BG3347	pCN-PDC1-GFP #2	pCN-PDC1-GFP	BG2	This work
Phagocytosis-induced promoters (*URA3*-marked)				
BG3322	pCU-ACO2 #1	*ura3*Δ::Tn903 G418^R^, pCU-ACO2	BG14	This work
BG3323	pCU-ACO2 #2	*ura3*Δ::Tn903 G418^R^, pCU-ACO2	BG14	This work
BG2978	pCU-ACO2-GFP #1	*ura3*Δ::Tn903 G418^R^, pCU-ACO2-GFP	BG14	This work
BG2979	pCU-ACO2-GFP #2	*ura3*Δ::Tn903 G418^R^, pCU-ACO2-GFP	BG14	This work
BG3324	pCU-LYS21 #1	*ura3*Δ::Tn903 G418^R^, pCU-LYS21	BG14	This work
BG3325	pCU-LYS21 #2	*ura3*Δ::Tn903 G418^R^, pCU-LYS21	BG14	This work
BG2982	pCU-LYS21-GFP #1	*ura3*Δ::Tn903 G418^R^, pCU-LYS21-GFP	BG14	This work
BG2983	pCU-LYS21-GFP #2	*ura3*Δ::Tn903 G418^R^, pCU-LYS21-GFP	BG14	This work
Phagocytosis-induced promoters (NAT^R^-marked)				
BG3334	pCN-ACO2 #1	pCN-ACO2	BG2	This work
BG3335	pCN-ACO2 #2	pCN-ACO2	BG2	This work
BG3348	pCN-ACO2-GFP #1	pCN-ACO2-GFP	BG2	This work
BG3349	pCN-ACO2-GFP #2	pCN-ACO2-GFP	BG2	This work
BG3336	pCN-LYS21 #1	pCN-LYS21	BG2	This work
BG3337	pCN-LYS21 #2	pCN-LYS21	BG2	This work
BG3350	pCN-LYS21-GFP #1	pCN-LYS21-GFP	BG2	This work
BG3351	pCN-LYS21-GFP #2	pCN-LYS21-GFP	BG2	This work
Nutritionally regulated promoters (*URA3*-marked)				
BG3326	pCU-MET3 #1	*ura3*Δ::Tn903 G418^R^, pCU-MET3	BG14	This work
BG3327	pCU-MET3 #2	*ura3*Δ::Tn903 G418^R^, pCU-MET3	BG14	This work
BG2512	pCU-MET3-GFP #1	*ura3*Δ::Tn903 G418^R^, pCU-MET3-GFP	BG14	This work
BG3340	pCU-MET3-GFP #2	*ura3*Δ::Tn903 G418^R^, pCU-MET3-GFP	BG14	This work
Nutritionally regulated promoters (NAT^R^-marked)				
BG3338	pCN-MET3 #1	pCN-MET3	BG2	This work
BG3339	pCN-MET3 #2	pCN-MET3	BG2	This work
BG3352	pCN-MET3-GFP #1	pCN-MET3-GFP	BG2	This work
BG3353	pCN-MET3-GFP #2	pCN-MET3-GFP	BG2	This work

NA, not applicable; NAT^R^, nourseothricin-resistant.

### Plasmid construction

All plasmids used in this study are listed in Table 2. The *URA3*-marked and NAT-marked plasmid backbones are derived from the pGRB2.1 and pBM16 plasmids, respectively (Frieman *et al.* 2002; Ma *et al.* 2009). We include a more complete description of their design here.

**Table 2:** Plasmids used in this study

Plasmid Name	Description	Parent Vector	Bacterial Stock	Source	Genbank Accession	Addgene ID
General use						
pGRB2.0	CEN/ARS plasmid [Ap^R^, *URA3*]	pRS406	b65	G. Rotano, unpublished	KF040394	45340
pGRB2.1	CEN/ARS plasmid containing HIS3 3′ UTR [Ap^R^, *URA3*]	pGRB2.0	b54	Frieman *et al.* 2002	KF040395	45341
pGRB2.2	PGK1pr on CEN/ARS plasmid also containing HIS3 3′ UTR [Ap^R^, *URA3*]	pGRB2.1	b162	Frieman *et al.* 2002	KF040396	45342
pGRB2.3	GFP driven by PGK1pr on CEN/ARS plasmid [Ap^R^, *URA3*]	pGRB2.2	b164	This work	KF040397	45343
pBM16	CEN/ARS plasmid containing NAT^R^ cassette [Ap^R^, NAT^R^]	pUC19	b1564	Ma *et al.* 2009	KF040398	45344
pBM16.1	CEN/ARS plasmid containing NAT^R^ cassette; same backbone as pBM16, with more limited MCS. [Ap^R^, NAT^R^]	pBM16	b1879	This work	KF040399	45345
Constitutive promoters						
pCU-EGD2	EGD2pr empty vector [Ap^R^, *URA3*]	pGRB2.1	b2448	This work	KF040370	45315
pCU-EGD2-GFP	EGD2pr-GFP [Ap^R^, *URA3*]	pGRB2.3	b2037	This work	KF040371	45316
pCN-EGD2	EGD2pr empty vector [Ap^R^, NAT^R^]	pBM16.1	b2454	This work	KF040372	45317
pCN-EGD2-GFP	EGD2pr-GFP [Ap^R^, NAT^R^ ]	pBM16	b2236	This work	KF040373	45318
pCU-HHT2	HHT2pr empty vector [Ap^R^, *URA3*]	pGRB2.1	b2449	This work	KF040374	45319
pCU-HHT2-GFP	HHT2pr-GFP [Ap^R^, *URA3*]	pGRB2.3	b2038	This work	KF040375	45320
pCN-HHT2	HHT2pr empty vector [Ap^R^, NAT^R^]	pBM16.1	b2455	This work	KF040376	45321
pCN-HHT2-GFP	HHT2pr-GFP [Ap^R^, NAT^R^]	pBM16	b2238	This work	KF040377	45322
pCU-PDC1	PDC1pr empty vector [Ap^R^, *URA3*]	pGRB2.1	b2450	This work	KF040378	45323
pCU-PDC1-GFP	PDC1pr-GFP [Ap^R^, *URA3*]	pGRB2.3	b2039	This work	KF040379	45324
pCN-PDC1	PDC1pr empty vector [Ap^R^, NAT^R^]	pBM16.1	b2456	This work	KF040380	45325
pCN-PDC1-GFP	PDC1pr-GFP [Ap^R^, NAT^R^]	pBM16	b2240	This work	KF040381	45326
Macrophage-induced promoters						
pCU-ACO2	ACO2pr empty vector [Ap^R^, *URA3*]	pGRB2.1	b2451	This work	KF040382	45327
pCU-ACO2-GFP	ACO2pr-GFP [Ap^R^, *URA3*]	pGRB2.3	b2230	This work	KF040383	45328
pCN-ACO2	ACO2pr empty vector [Ap^R^, NAT^R^]	pBM16.1	b2457	This work	KF040384	45329
pCN-ACO2-GFP	ACO2pr-GFP [Ap^R^, NAT^R^]	pBM16	b2235	This work	KF040385	45330
pCU-LYS21	LYS21pr empty vector [Ap^R^, *URA3*]	pGRB2.1	b2452	This work	KF040386	45331
pCU-LYS21-GFP	LYS21pr-GFP [Ap^R^, *URA3*]	pGRB2.3	b2232	This work	KF040387	45332
pCN-LYS21	LYS21pr empty vector [Ap^R^, NAT^R^]	pBM16.1	b2458	This work	KF040388	45334
pCN-LYS21-GFP	LYS21pr-GFP [Ap^R^, NAT^R^]	pBM16	b2239	This work	KF040389	45335
Nutritionally regulated promoters						
pCU-MET3	MET3pr empty vector [Ap^R^, *URA3*]	pGRB2.1	b2453	This work	KF040390	45336
pCU-MET3-GFP	MET3pr-GFP [Ap^R^, *URA3*]	pGRB2.3	b1971	This work	KF040391	45337
pCN-MET3	MET3pr empty vector [Ap^R^, NAT^R^]	pBM16.1	b2459	This work	KF040392	45338
pCN-MET3-GFP	MET3pr-GFP [Ap^R^, NAT^R^]	pBM16	b2410	This work	KF040393	45339

UTR, untranslated region; NAT^R^, nourseothricin-resistant.

The pGRB vectors were created by cloning a chimeric *C. glabrata* CEN/ARS sequence into pRS406 (Sikorski and Hieter 1989), which was linearized via *Aat*II restriction digestion, creating pGRB2.0. The CEN sequence was isolated from *C. glabrata* strain BG2 centromere H, based on previously identified *C. glabrata* centromeric sequences (Kitada *et al.* 1996). The ARS sequence was functionally isolated from *C. glabrata* strain BG2 and corresponds to nucleotides 286060-286210 of chromosome F in the published *C. glabrata* CBS138 sequence. The *HIS3* 3′ untranslated region was amplified from *C. glabrata* strain BG2 using polymerase chain reaction (PCR) and cloned into pGRB2.0 as a *Xho*I-*Kpn*I fragment, creating pGRB2.1. The *S. cerevisiae PGK1* promoter was amplified from *S. cerevisiae* using PCR and cloned into pGRB2.1 as a *Sac*I-*Xba*I fragment, creating pGRB2.2 (Domergue *et al.* 2005). The plasmid pGRB2.3 contains yEGFP3 (Cormack *et al.* 1997) downstream of the *PGK1* promoter, cloned as a *Eco*RI and *Sal*I fragment.

The basic NAT^R^ backbone for use in *C. glabrata* is pBM16 (Ma *et al.* 2009). The NAT^R^ cassette was generated by PCR-amplifying Sc*TEF1*p and the *NAT1* open reading frame and subcloning them into pRS416 (Sikorski and Hieter 1989); the resulting Sc*TEF1*p–*NAT1*–*ScCYC1 3′UTR* cassette was isolated as a *Sac*I/*Kpn*I fragment, blunt-ended, and subcloned into the *Nde*I site in the pUC19 backbone, which itself had been blunt-ended. The *C. glabrata* CEN/ARS sequence was PCR-amplified from pGRB2.0 using primers that contained *Aat*II restriction sites and then subcloned into the unique *Aat*II site in the pUC19 backbone, thus creating pBM16. pBM16.1 is the same as pBM16, except its multiple cloning site (MCS) was replaced with an oligonucleotide containing *Sac*I and *Kpn*I sites, to facilitate cloning of *Sac*I/*Kpn*I fragments from pGRB2.0-derived vectors

**Figure 1:**
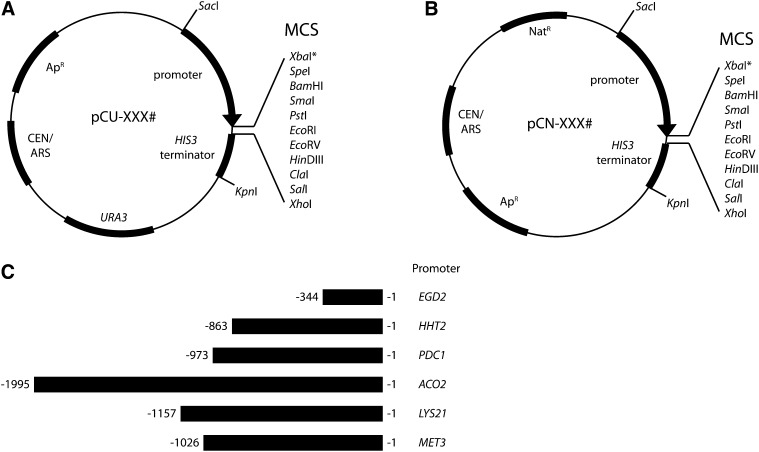
Schematic diagrams of the pCU and pCN series of *C. glabrata* vectors. Naming convention was chosen to indicate the plasmids have a CEN/ARS and either *URA3* (pCU; A) or nourseothricin resistance (NAT^R^) (pCN; B) cassettes for selection in *C. glabrata*. Both backbones carry an Ap^R^ gene and pMB1 (ColEI family) origin (not shown) for growth and selection in *E. coli*. The restriction sites in the multiple cloning site (MCS) between the promoter and terminator are listed. *The *Xba*I site is not present in the MCS of pCU-LYS21 or pCN-LYS21. Refer to Table 4 to determine which MCS restriction sites are unique in each plasmid, because this depends on the particular backbone and promoter combination. XXX# is a placeholder to designate the name of the promoter present in a particular vector. (C) The lengths of the promoters cloned into the pCU and pCN vectors using *Sac*I-*Xba*I restriction digestion for all constructs except *LYS21*, which used *Sac*I-*Spe*I, thereby eliminating the *Xba*I site in the MCS, are depicted.

Primers used to amplify each *C. glabrata* promoter during plasmid construction are listed in Table 3. They contain restriction sites to facilitate cloning; all forward primers contain a *Sac*I site and reverse primers contain *Spe*I (for *LYS21*pr) or *Xba*I sites (all others). The *MET3* promoter was amplified from BG2 genomic DNA; all other promoters were amplified from CBS138 genomic DNA. The PCR products were TOPO-cloned (Invitrogen) and sequenced. Empty CEN/ARS *URA3*-marked vectors (pCU series; Figure 1A) were created by subcloning each promoter using *Sac*I/*Spe*I (for *LYS21*) or *Sac*I/*Xba*I (all others) and ligating them into pGRB2.1 cut with *Sac*I/*Xba*I. The *LYS21* promoter contains an internal *Xba*I site, so *Spe*I was used for subcloning; this eliminates *Xba*I from the MCS of the *LYS21* vectors. The NAT-marked versions of the empty vectors (pCN series; Figure 1) were created by cloning each pCU series plasmid with *Sac*I/*Kpn*I to release the promoter-MCS-terminator fragment and ligating this into pBM16.1. These NAT-marked empty vectors are the pCN series of plasmids (Figure 1B). pCU-ACO2 was subcloned to the pBM16.1 vector using a *Sac*I/partial *Kpn*I digestion, because the *ACO2* promoter contains a *Kpn*I site.

**Table 3:** Primers used in this study

Oligo Number	Description	Sequence (5′->3′)
NAT^R^ cassette		
2540	ScTEF1p, for	caaggagctcCATAGCTTCAAAATGTTTCTACTCC
2541	ScTEF1p, rev	cgcggatccAAAACTTAGATTAGATTGCTATGC
2542	NAT1 orf, for	ctagagatctAAAATGGGCACCACTCTTGACG
2543	NAT1 orf, rev	acgcgtcgacTTAGGGGCAGGGCATGCTCATG
pBM16.1 MCS		
	MCS, for	aattgagagctcgaccatcaagggtaccttgca
	MCS, rev	aggtacccttgatggtcgagctctc
Constitutive promoters		
4634	*EGD2*p, for	aaaagagctcTGTCCACTTCACTCACCAGT
4636	*EGD2*p, rev	aaaatctagaCTTTGTATATCTGTATTATTG
4637	*HHT2*p, for	aaaagagctcTGTTATTGATTATTTATTTATTTG
4638	*HHT2*p, rev	aaaatctagaTATGTATGTGTTGTGTTTTG
4639	*PDC1*p, for	aaaagagctcAGCATTTTTATACACGTTTTAC
4640	*PDC1*p, rev	aaaatctagaTGTTAATGTTTTTTGGCAATTG
Macrophage-induced promoters		
5114	*ACO2*p, for	attagagctcCTGCAGTGTCCCGTTTGTTTC
5115	*ACO2*p, rev	attatctagaTGCTAGTGGGTACGAATTGTAG
5120	*LYS21*p, for	attagagctcAGAAAGCGAAGAAGATATATC
5121	*LYS21*p, rev	attaactagtCTTTAATATTCTTTGTTCAGC
Nutritionally regulated promoters		
4036	*MET3*p, for	cttgagagctcATACCAGTTACAATTAGTATTACAATGGTTTAC
4035	*MET3*p, rev	gctctagaTTGTTAGGTGTTTCTTTTCTGGAGTGTTA
qRT-PCR primers		
5980	GFP, for	CCACTCAATCTGCCTTATC
5981	GFP, rev	ATCCATACCATGGGTAATAC
5883	*TUB1*, for	GGTGATGTGGTAACAAGAGATG
5884	*TUB1*, rev	GTAGCAGATACCGATCTTGAAAC
6426	*TUB1*, rev 2	TCGTAGCAGATACCGATCTTGAA
6597	Ap^R^ gene, for	AAGCCATACCAAACGACGAG
6598	Ap^R^ gene, rev	TTGCCGGGAAGCTAGAGTAA

The DNA sequences of primers used in this article are listed. Capital letters in the DNA sequence hybridize to the target sequence; linkers and restrictions sites added are written in lowercase letters. NAT^R^, nourseothricin-resistant; for, forward; rev, reverse; qRT-PCR, quanitative reverse-transcription polymerase chain reaction.

Each promoter also was subcloned into pGRB2.3 using *Sac*I/*Xba*I or *Sac*I/*Spe*I restriction digestions, positioning the promoter upstream of the MCS and GFP open reading frame, creating a GFP reporter plasmid. To construct NAT-marked versions of the GFP reporter strains, the promoter-MCS-GFP-terminator fragment was cut from the pGRB backbone using *Sac*I/*Acc*65I restriction digestions and ligated into pBM16. pBM16 has *E. coli* origin of replication and selectable markers, a *C. glabrata* CEN/ARS, and a NAT resistance (NAT^R^) cassette.

All plasmids have been deposited with corresponding sequences at Addgene. Addgene and GenBank accession numbers are included in Table 2.

### Plasmid copy number

Total DNA was isolated from *C. glabrata* strains carrying pCU or pCN series plasmids. Log-phase cultures of *C. glabrata* strains were pelleted by centrifugation and washed in a solution of 50 mM Tris (pH 8.0), 10 mM EDTA, and 50 mM β-mercaptoethanol. The cells were resuspended in spheroplasting buffer [1.1 M sorbitol, 50 mM Tris (pH 8.0), 10 mM EDTA] supplemented with 1 mM β-mercaptoethanol. Yeast lytic enzyme (#152270; ICN Biomedicals) was added and the cells were incubated at 30°C for 10 min. Spheroplasts were collected by centrifugation and washed twice in spheroplasting buffer. DNA was isolated from the washed spheroplasts using the MasterPure Yeast DNA Purification Kit (#MPY80200; Epicentre). Total DNA was treated with RNase and cleaned by phenol/chloroform extraction and ethanol precipitation. Total DNA was then used in a quantitative PCR (qPCR) reaction to assess relative amounts of plasmid and genomic DNA. Plasmid DNA was monitored by amplifying a portion of the Ap^R^ gene (primers 6596 and 6598); genomic DNA was monitored by amplifying a portion of the *TUB1* gene (primers 5883 and 6426) (Table 3 shows primer sequences). Average cycle threshold values for experimental samples were compared with a standard curve of genomic DNA from strain BG2389 to generate relative starting quantities of DNA. Strain BG2389 has a single copy of the Ap^R^ gene integrated into the genome and is expected to have Ap^R^ and *TUB1* in equal copy number. Quantitative PCR was performed in technical triplicate on each sample with each primer set. The average Ap^R^ and *TUB1* signal was calculated for each strain, and plasmid copy number was calculated as Ap^R^/*TUB1*. Two independent yeast strains carrying a given plasmid were monitored; the plasmid copy number in replicate strains was averaged as shown in Table S2.

### Rate of plasmid loss

Yeast strains were grown to mid-log phase under selection in SD-Ura or YPD+NAT media for pCU or pCN plasmids, respectively. Cultures were washed twice in sterile water and diluted to an optical density (OD) of OD_600_ = 0.02 in YPD (nonselective) media (t = 0) and grown at 30°C. After 5 hr, an OD_600_ measurement was taken to calculate the number of times the culture had doubled, and then culture was diluted back to OD_600_ = 0.02 to keep it in log phase at 30°C. At t = 10 hr, the OD_600_ of the culture was again measured and cells were plated onto YPD (3 plates/media/strain) at approximately 200 cells/plate. Plates were grown at 30°C for 1–2 days and then were replica-plated onto selective media (SD−Ura or YPD+NAT). After 1 day of growth at 30°C, the selective plates were scored for colonies having lost the plasmid (Ura^-^ or NAT^S^) or having retained the plasmid (any portion of the colony is Ura^+^ or NAT^R^). At each time point, the fraction of the population that contained the plasmid on the original YPD plates was calculated from the selective plates as CFU_growth_/CFU_total_. The number of generations between time points was the sum of the number of generations from t = 0 to t = 5, and then from t = 5 to t = 10, using the formula [#gen = log_2_(OD_t2_/OD_t1_)]. The rate of plasmid loss per generation was calculated as:

1-{[(fraction of the population with plasmid at t=10h)/(fraction of population with plasmid at t=0h)]^(1/generations)}.

### Plasmid integration into genome

Two *C. glabrata* yeast strains carrying pCU-PDC1 were grown to saturation in liquid SD−Ura media. Cultures were plated onto 10 SD−Ura plates each, at a density of approximately 100 colonies per plate and grown 2 days at 30°C. These colonies were then replica-plated onto 5-FOA media and grown overnight at 30°C. Each colony on the original SD−Ura plate was monitored to determine if it was sensitive or resistant to 5-FOA. If the pCU-PDC1 plasmid had integrated into the genome, we would have anticipated a 5-FOA^S^ phenotype (Castano *et al.* 2003), whereas cells in which the pCU plasmid is maintained as an episome show a 5-FOA^R^ phenotype, reflecting plasmid loss.

### Flow cytometry

Cells from liquid cultures were washed twice in sterile PBS and resuspended in 0.5 ml PBS. A FACScalibur flow cytometer (BD Biosciences) was used to measure GFP fluorescence. Detector levels were adjusted such that a nonfluorescent strain had a median fluorescence of approximately 10. Populations also were gated based on forward and side scatter of the nonfluorescent strain. For each sample, data for 10^4^ gated cells were collected using CellQuest Pro software (BD Biosciences). Further statistical analysis and graphical representation were performed using FloJo software. Fluorescence was measured from two independent strains carrying each construct. “Stationary” samples were taken from cultures grown overnight in appropriate media. The overnight cultures were diluted 1:20 and grown at 30°C for 4 hr, to OD_600_ of approximately 0.3, and used as the “exponential” samples.

### Phagocytosis assays and *C. glabrata* isolation

Phagocytosis assays were performed as previously described, with some alterations (Kaur *et al.* 2007). Murine macrophage-like cell line J774A.1 cells were seeded into 15-cm-diameter tissue culture (TC) dishes (BD353025; BD Bioscience) and grown to confluence in DMEM plus fetal bovine serum (FBS) plus P/S (DMEM supplemented with 10% FBS, 100 U/ml penicillin, and 100 μg/ml streptomycin; 120-095-72; Quality Biologicals). Log-phase *C. glabrata* cells were added to the J477A.1 cells at a multiplicity of infection of 3 and incubated at 37°C plus 5% CO_2_ for 1 hr to allow the yeast to settle onto the J477A.1 cells. After incubating for 1 hr, the non-cell-associated yeast were removed by aspiration and washing in DMEM. The J477A.1 and *C. glabrata* were coincubated for an additional 2 hr. After 2 hr, media was decanted and the macrophages were scraped from the TC dishes and lysed in 10 ml cold DEPC dH_2_O supplemented with RNaseA (Fermentas EN0531) and RNaseA/T1 (Fermentas EN0551) to digest mammalian RNA. *C. glabrata* released from the J774A.1 cells were collected by centrifugation (4000 rpm, 4°C, 10 min). The *C. glabrata* cells were then washed twice in cold DEPC dH_2_O containing RNase inhibitors (Protect RNA, Sigma R7397) and spun as described previously. Finally, the *C. glabrata* cells were resuspended in 1 ml DEPC dH_2_O plus Protect RNA and transferred to 2-ml screw-cap tubes that contained approximately 500 μl acid-washed glass beads. The cells were spun in a microfuge (4000 rpm, 4°C, 5 min). The supernatant accessible above the glass beads was aspirated and the yeast were frozen in liquid nitrogen and stored at −80°C until RNA isolation commenced.

To control for growth in DMEM and TC conditions, *C. glabrata* cells also were inoculated into TC dishes with DMEM supplemented with FBS and P/S without J774A.1 and grown for a total of 3 hr at 37°C plus 5% CO_2_. The contents of the dish were transferred to a 50-ml conical tube and centrifuged (4000 rpm, 4°C, 10 min). The pelleted yeast were washed in DEPC dH_2_O supplemented with Protect RNA, transferred to a screw-cap tube, and pelleted with glass beads as described.

### Quantitative reverse-transcription PCR to monitor promoter strength

RNA was isolated from *C. glabrata* (phagocytosed or media-grown controls) using a guanidine thiocyanate mixture (4 M guanidine thiocyanate, 2% β-ME) lysis and acid phenol extraction; 0.4 ml guanidine thiocyanate mixture was added to each frozen pellet and cells were disrupted using a Fast-Prep (Bio101 Thermo Savant). Lysates were transferred to a new nuclease-free tube and RNA was recovered by performing two acid phenol/chloroform extractions. The extracted RNA was then precipitated and resuspended in DEPC dH_2_O. The RNA was treated with DNase (NEB B0303S) to remove any residual genomic DNA, and then the acid phenol extraction and ethanol precipitation were performed again. Reverse-transcription of purified RNA was performed linearly with Superscript III (Invitrogen). Quantitative PCR was performed in triplicate, monitoring EvaGreen fluorescence in a BioRad Thermocycler. Relative GFP expression for each strain is calculated as the average GFP signal normalized to the average *TUB1* values (primers 5883 and 5884). Figure 4 shows the relative expression for each strain, averaged across two biological replicates of the macrophage infection.

### Phagocytosis assays and microscopic imaging

*C. glabrata* strains carrying pCU-ACO2-GFP or pCU-LYS21-GFP plasmids were grown to OD of 7.8–9.4 in SD-Ura media. The cells were washed in PBS and resuspended at a density of 4×10^6 cells/ml in DMEM supplemented with FBS and P/S. J774A.1 cells were seeded into 24-well tissue culture plates (Beckman Dickinson 353047) with or without sterile glass coverslips in each well. Once the J774A.1 had grown to confluence, they were washed with fresh DMEM, which was then aspirated from the wells. Each well was then filled with 100 μl yeast cells in DMEM supplemented with FBS and P/S, which is effectively a multiplicity of infection of 1 (estimating 4×10^5^ macrophages/well). After a 1-hr incubation at 37°C plus 5% CO_2_, during which the yeast settled onto the macrophage and were engulfed, the media and unbound yeast were aspirated from the wells. The J774A.1 cells were washed twice with DMEM to remove any unbound yeast and 500 μl DMEM supplemented with FBS and P/S was added to each well. The yeast and J774A.1 cells were coincubated for an additional 2 hr. To stop the infection, the media was aspirated from the wells with coverslips and the J774A.1 and *C. glabrata* were fixed in a 1% formaldehyde solution for 5 min. The coverslips were then washed in PBS and then mounted onto glass slides with Vectashield. In parallel, 100 μl yeast cells were seeded into 24-well plates without J774A.1 cells to control for growth conditions and were allowed to grow for a total of 3 hr. These yeast were pelleted in microfuge tubes, media was aspirated, and the cells were resuspended and fixed in 1% formaldehyde solution for 5 min. The fixed unphagocytosed yeast were mounted onto glass slides without Vectashield. All samples were imaged on a Zeiss Axioskop microscope at 100× objective and bright field differential image contrast and fluorescent images were captured.

## Results

### Plasmid design and construction

The plasmids described here were designed as tools for molecular genetic experiments in *C. glabrata*. Promoters of six genes were chosen and cloned into *URA3*-marked or NAT^R^-marked episomal plasmids. In addition to making “empty” vectors suitable for cloning any desired gene under the control of each promoter, reporter constructs were created where GFP was cloned into each set of plasmids so that the relative strength of each promoter could be measured by monitoring GFP abundance.

Three “constitutive” promoters were chosen from unpublished data analyzing transcriptional profiles of *C. glabrata* cells growing in YPD in exponential phase or stationary phase. These promoters, *EGD2* (CAGL0M07161g), *HHT2* (CAGL0C04114g), and *PDC1* (CAGL0M07920g), were chosen as representatives of low-level, medium-level, and high-level expression during both exponential phase and stationary phase growth. Using transcriptional microarray data comparing *C. glabrata* expression in RPMI and after phagocytosis by the macrophage-like mammalian cell line J774A.1 (Kaur *et al.* 2007), we identified promoters with a high macrophage/RPMI ratio. To identify genes specifically induced after phagocytosis, rather than by starvation, we selected genes that were not strongly induced in the stationary phase, as compared with the exponential phase, when grown in laboratory media (B. Green, unpublished data). From this analysis, we chose *ACO2* (CAGL0F02431g) and *LYS21* (CAGL0J09240g) as phagocytosis-induced genes. Finally, the *MET3* (CAGL0B03839g) promoter was developed as a regulatable promoter based on similar constructs used in *S. cerevisiae* and *Candida albicans* (Stark 1998; Care *et al.* 1999).

For most genes, we used PCR amplification to isolate and clone the promoter (the complete intergenic region upstream of the designated gene). For the *MET3* promoter, we amplified 1026 bp immediately upstream of the *MET3* open reading frame. The length of each cloned promoter is indicated in Figure 1C.

Ultimately, each promoter was cloned into four plasmid backbones for use in *C. glabrata*. The backbones contain a *URA3* auxotrophic marker or a dominant NAT^R^ cassette, as well as a multiple cloning site or GFP downstream of the promoters. Plasmids containing the *C. glabrata* CEN/ARS and the *URA3* auxotrophic marker are designated as the pCU series (Figure 1A). They are derived from the pGRB plasmid backbone, which contains a *S. cerevisiae URA3* cassette to serve as an auxotrophic marker and functions for selection in *C. glabrata*. The pCU plasmids also contain an Ap^R^ gene and F1, M13, and pMB1 (ColE1 family) origins for growth and selection in *E. coli*. The pCN series of plasmids have the *C. glabrata* CEN/ARS and a NAT^R^ cassette for selection and maintenance in *C. glabrata* (Figure 1B). The pCN plasmids are derived from pBM16.1, which has an Ap^R^ gene and a pMB1 (ColE1 family) origin for growth and selection in *E. coli*. Plasmid names and descriptions are indicated in Table 3.

The “empty” versions of each pCU and pCN series plasmid contain an MCS immediately downstream of the promoter, followed by a *HIS3* terminator. These vectors are suitable for further subcloning, placing any gene of interest under the control of the given promoter. Table 4 lists the restriction enzyme recognition sites found in the MCS for each plasmid and indicates which sites are unique for each construct. Additionally, we made versions of each plasmid that have GFP placed under the control of each promoter. These reporter plasmids were used to assess the strength of each promoter through monitoring GFP expression levels.

**Table 4:** Restriction sites found in the multiple cloning sites of the pCU and pCN plasmids

	Promoter	MCS											Other Sites	
pCU series	*EGD2*	***Xba*I**	***Spe*I**	***Bam*HI**	***Sma*I**	*Pst*I	***Eco*RI**	*Eco*RV	*Hind*III	*Cla*I	*Sal*I	***Xho*I**	***Sac*I**	***Kpn*I**
	*HHT2*	***Xba*I**	***Spe*I**	***Bam*HI**	***Sma*I**	*Pst*I	*Eco*RI	*Eco*RV	*Hind*III	*Cla*I	*Sal*I	***Xho*I**	***Sac*I**	***Kpn*I**
	*PDC1*	***Xba*I**	***Spe*I**	*Bam*HI	***Sma*I**	*Pst*I	***Eco*RI**	*Eco*RV	*Hind*III	*Cla*I	***Sal*I**	***Xho*I**	***Sac*I**	***Kpn*I**
	*ACO2*	***Xba*I**	*Spe*I	*Bam*HI	***Sma*I**	*Pst*I	*Eco*RI	*Eco*RV	*Hind*III	*Cla*I	***Sal*I**	*Xho*I	***Sac*I**	*Kpn*I
	*LYS21*		***Spe*I**	*Bam*HI	***Sma*I**	*Pst*I	***Eco*RI**	*Eco*RV	*Hind*III	*Cla*I	*Sal*I	***Xho*I**	***Sac*I**	***Kpn*I**
	*MET3*	***Xba*I**	***Spe*I**	*Bam*HI	***Sma*I**	*Pst*I	***Eco*RI**	*Eco*RV	*Hind*III	*Cla*I	***Sal*I**	***Xho*I**	***Sac*I**	***Kpn*I**
pCN series	*EGD2*	***Xba*I**	***Spe*I**	***Bam*HI**	*Sma*I	***Pst*I**	***Eco*RI**	***Eco*RV**	*Hind*III	*Cla*I	*Sal*I	***Xho*I**	***Sac*I**	***Kpn*I**
	*HHT2*	***Xba*I**	***Spe*I**	***Bam*HI**	*Sma*I	*Pst*I	*Eco*RI	***Eco*RV**	*Hind*III	*Cla*I	*Sal*I	***Xho*I**	***Sac*I**	***Kpn*I**
	*PDC1*	***Xba*I**	***Spe*I**	*Bam*HI	*Sma*I	*Pst*I	***Eco*RI**	***Eco*RV**	*Hind*III	*Cla*I	***Sal*I**	***Xho*I**	***Sac*I**	***Kpn*I**
	*ACO2*	***Xba*I**	*Spe*I	*Bam*HI	*Sma*I	*Pst*I	*Eco*RI	*Eco*RV	*Hind*III	*Cla*I	***Sal*I**	*Xho*I	***Sac*I**	*Kpn*I
	*LYS21*		***Spe*I**	*Bam*HI	*Sma*I	***Pst*I**	***Eco*RI**	***Eco*RV**	*Hind*III	*Cla*I	*Sal*I	***Xho*I**	***Sac*I**	***Kpn*I**
	*MET3*	***Xba*I**	***Spe*I**	*Bam*HI	*Sma*I	*Pst*I	***Eco*RI**	***Eco*RV**	*Hind*III	*Cla*I	***Sal*I**	***Xho*I**	***Sac*I**	***Kpn*I**

Bold lettering indicates the sequence is unique in that particular plasmid. Sites listed under the MCS heading are found in the multiple cloning site of the plasmid; *Sac*I and *Kpn*I are located at the beginning of the promoter and end of the *HIS3* terminator, respectively. MCS, multiple cloning site.

### Plasmid maintenance

Three aspects of these plasmids were monitored to characterize plasmid maintenance in *C. glabrata*: the copy number, the rate of plasmid loss, and the rate at which the plasmids integrate into the genome. Copy number was measured using qPCR to amplify the Ap^R^ gene (from the plasmid backbone) and *TUB1* (from the genome) from total DNA isolated from select yeast strains. *C. glabrata* strains carrying pCU-EGD2, pCN-EGD2, pCU-ACO2, or pCN-ACO2 were chosen because these constructs contain the shortest (*EGD2*) and longest (*ACO2*) promoters in both backbones. As shown in Table S2, the pCU vectors are present at approximately 2 copies per cell. The pCN vectors are present at 0.6 to 0.8 copies per cell.

Plasmid stability was measured by growing four *C. glabrata* strains carrying pCU-PDC1 or pCN-PDC1 (2 strains per construct) nonselectively for 10 hr and determining how many cells retain the plasmid by plating on selective media. We estimate pCU-PDC1 plasmids were lost at a rate of 4.6% per generation, and pCN-PDC1 plasmids were lost at a rate of 6.55% per generation (Table S3).

Finally, we wanted to ensure that the pCU and pCN plasmids do not integrate into the genome. Two *C. glabrata* strains carrying pCU-PDC1 were grown selectively in SD−Ura media to saturation and then plated to SD−Ura. The SD−Ura plates were replica-plated to plates containing 5-FOA to counterselect any colonies that contained the *URA3* gene. Any 5-FOA^S^ colonies would represent cells that had integrated the *URA3* gene (and presumably the entire plasmid) into the genome. Conversely, 5-FOA^R^ cells represent those with *URA3* on an episomal plasmid, which may be lost and permit growth on 5-FOA. No 5-FOA^S^ colonies were detected in either strain tested, suggesting rates of integration into the genome are <0.04% (Table S4).

### Expression from *C. glabrata* promoters

**Figure 2:**
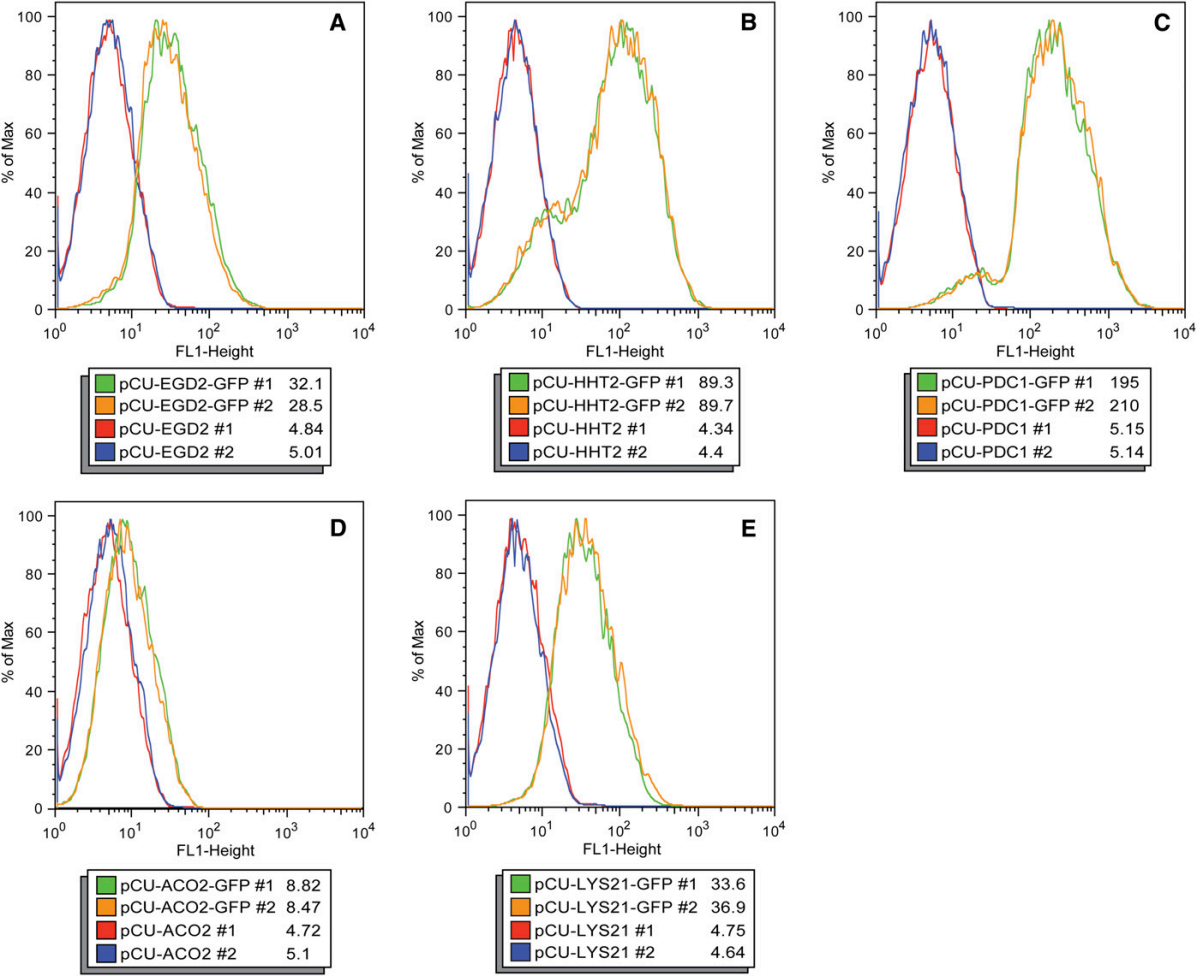
Fluorescence of exponential phase *C. glabrata* strains carrying pCU series plasmids. Flow cytometry was used to measure fluorescence levels in *C. glabrata* strains carrying pCU series plasmids containing *EGD2* (A), *HHT2* (B), *PDC1* (C), *ACO2* (D), and *LYS21* (E) promoters. The strains were diluted from a saturated overnight culture and grown to OD_600_ of approximately 0.3 before fluorescence was measured. Each panel depicts histograms of fluorescence for two *C. glabrata* strains containing the empty vectors (red and blue lines) and two *C. glabrata* strains containing GFP reporter vectors (green and orange lines) for a given promoter. The median fluorescence value for each strain population is shown.

**Figure 3:**
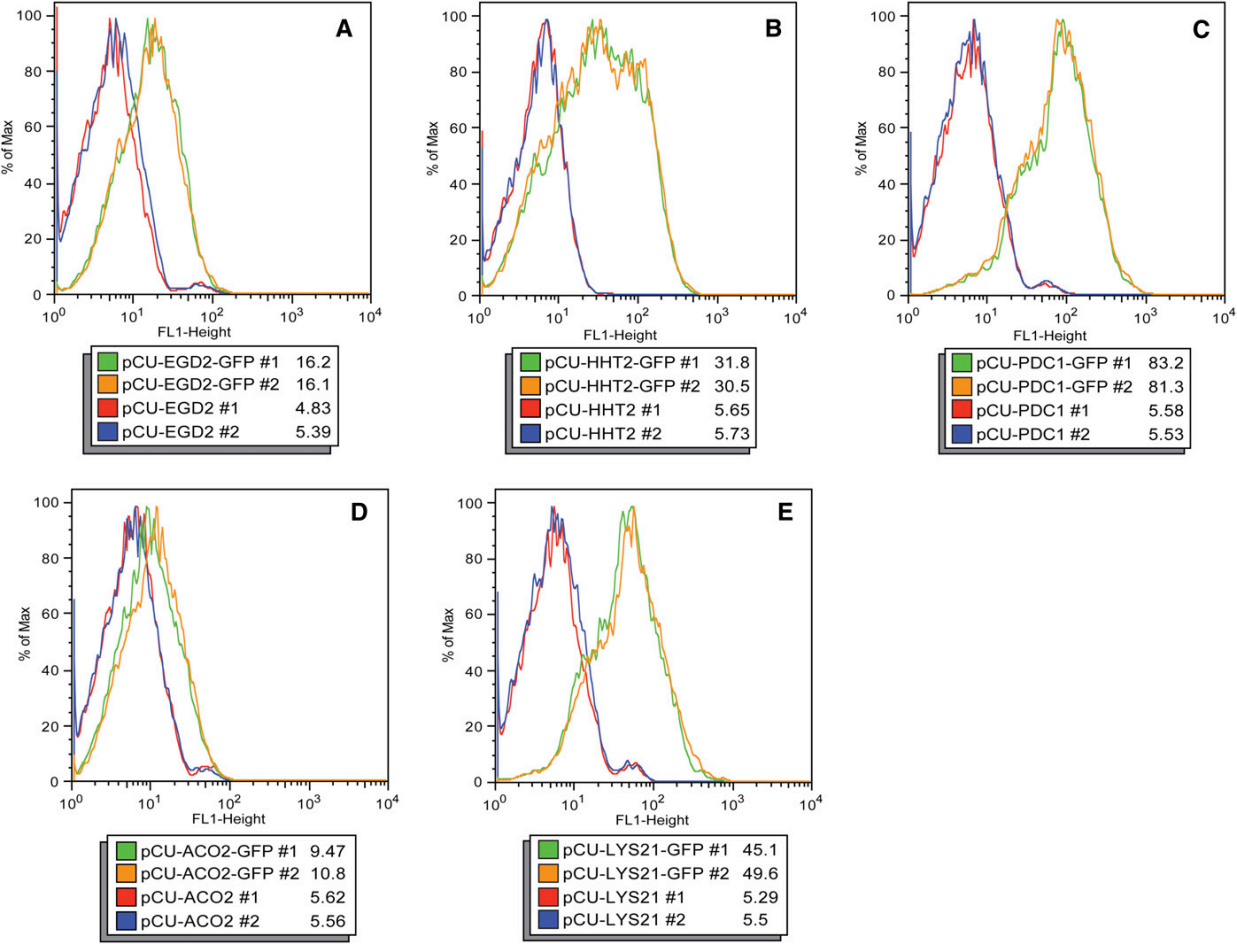
Fluorescence of stationary phase *C. glabrata* strains carrying pCU series plasmids. Flow cytometry was used to measure fluorescence levels in *C. glabrata* strains carrying pCU series plasmids containing *EGD2* (A), *HHT2* (B), *PDC1* (C), *ACO2* (D), and *LYS21* (E) promoters. Fluorescence of saturated overnight cultures was measured. Each panel depicts histograms of fluorescence for two *C. glabrata* strains containing the empty vectors (red and blue lines) and two *C. glabrata* strains containing GFP reporter vectors (green and orange lines) for a given promoter. The median fluorescence value for each strain population is shown.

The relative strength of each promoter was analyzed by monitoring GFP expression in *C. glabrata* strains carrying each plasmid construct. Fluorescence in strains carrying the pCU series of plasmids growing exponentially in SD−Ura was measured by flow cytometry (Figure 2). For each promoter construct, two *C. glabrata* strains carrying the empty vector and two strains carrying the GFP reporter plasmid were tested. Comparing the median fluorescence for the populations in Figure 2, A–C, the flow cytometry indicates expression from the pCU-EGD2-GFP is the lowest (median ∼30), followed by *HHT2* (median ∼89), and strains carrying pCU-PDC1-GFP had the highest level of fluorescence (median ∼202). This relationship of increasing strength of promoters (*EGD2*<*HHT2*<*PDC1*) is also true in stationary cultures, but expression from all strains is lower than what is observed in the corresponding log-phase cultures (Figure 3). In Figure 2, the histograms for strains carrying pCU-HHT2-GFP and pCU-PDC1-GFP have shoulders, possibly reflecting cells that have lost the plasmid or alternatively were delayed in exiting stationary phase.

The expression driven by the *ACO2* and *LYS21* phagocytosis-induced promoters also was assessed in standard laboratory media to assess the basal levels of expression from the *ACO2* and *LYS21* promoters during laboratory culture. To this end, *C. glabrata* strains carrying the pCU-ACO2-GFP and pCU-LYS21-GFP plasmids were grown in SD−Ura media and fluorescence was assessed using flow cytometry (Figure 2, D and E). As seen in Figure 2D, the *ACO2* promoter has extremely low basal expression in exponentially growing *C. glabrata* cells in SD−Ura media. The *LYS21* promoter has low basal expression in laboratory media, on par with the levels seen using the constitutive promoter *EGD2* (Figure 2, E and A).

Similar results were seen for *C. glabrata* strains carrying the pCN series of plasmids when grown in YPD+NAT media (data not shown).

### Expression after phagocytosis

**Figure 4:**
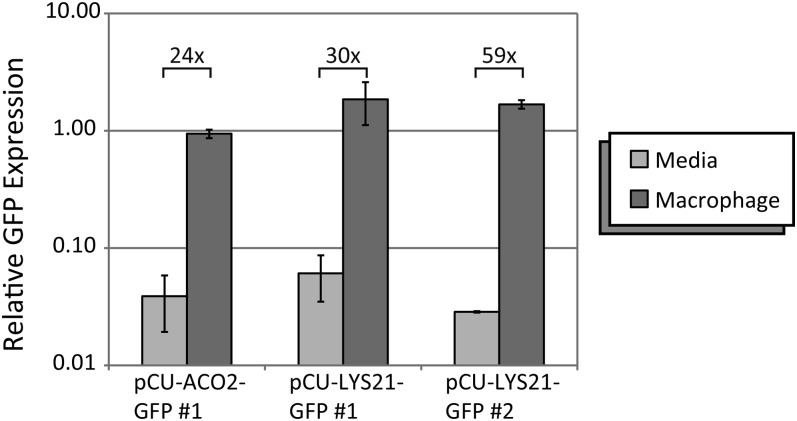
Quantitative reverse-transcription PCR (qRT-PCR) results of GFP expression during phagocytosis. qRT-PCR assessment of relative expression of *GFP* (normalized to *TUB1*) in *C. glabrata* grown in media or after phagocytosis by J774A.1 macrophage-like cells. The data are averages of two biological replicates (qPCR performed in triplicate) for each strain; error bars indicate the SD between the averages. The numbers above the bars indicate the average fold-change in *GFP* expression (normalized to *TUB1*) in *C. glabrata* that have been phagocytosed by J774A.1 (macrophage) *vs.* growth in tissue culture (TC) media (media). Data are shown for one *C. glabrata* strain carrying pCU-ACO2-GFP and two independent strains carrying pCU-LYS21-GFP.

**Figure 5:**
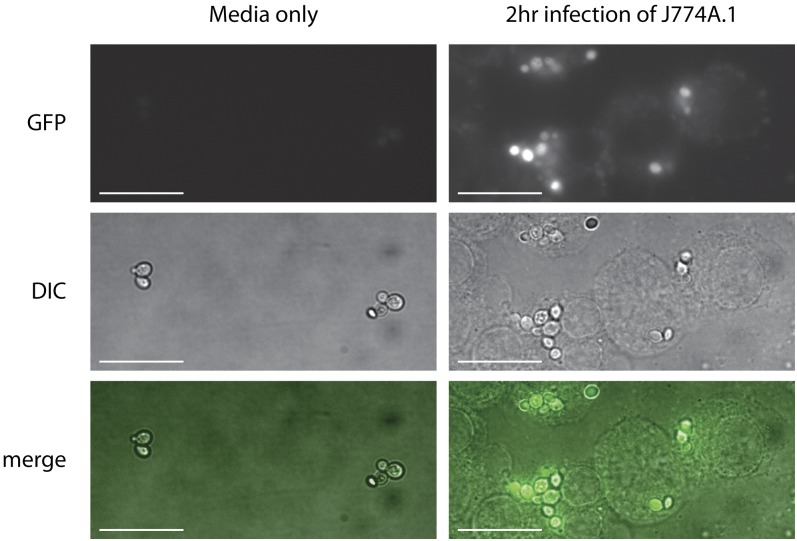
Expression from pCU-LYS21-GFP is increased in phagocytosed *C. glabrata. C. glabrata* strains carrying pCU-LYS21-GFP were grown in media only (left panels) or used to infect J774A.1 macrophage-like cells (right panels). After 2 hr, the cells were fixed and GFP expression was monitored by bright-field differential image contrast (DIC) and fluorescence microscopy. Merge panels were created by combining the bright-field and pseudo-colored GFP images using ImageJ. Scale bar is 10 μm.

The *C. glabrata* strains carrying plasmids with the phagocytosis-induced *ACO2* and *LYS21* promoters were used to infect J774A.1 cells and expression from the promoters was assessed by measuring GFP transcript levels using qRT-PCR. *C. glabrata* cells were isolated from J774A.1 cells after 2 hr of infection and GFP expression was measured (as normalized to *TUB1*). To control for growth in tissue culture media and conditions, *C. glabrata* also were grown in DMEM supplemented with FBS and P/S for an equivalent amount of time and GFP expression was monitored. As seen in Figure 4, expression of GFP from the pCU-ACO2-GFP or pCU-LYS21-GFP plasmids increased markedly in *C. glabrata* strains that had been phagocytosed by J774A.1 macrophage-like cells. Increased GFP expression also can be observed by fluorescence microscopy of phagocytosed *C. glabrata* cells (Figure 5).

### Nutritional regulation of the *MET3* promoter in *C. glabrata*

**Figure 6:**
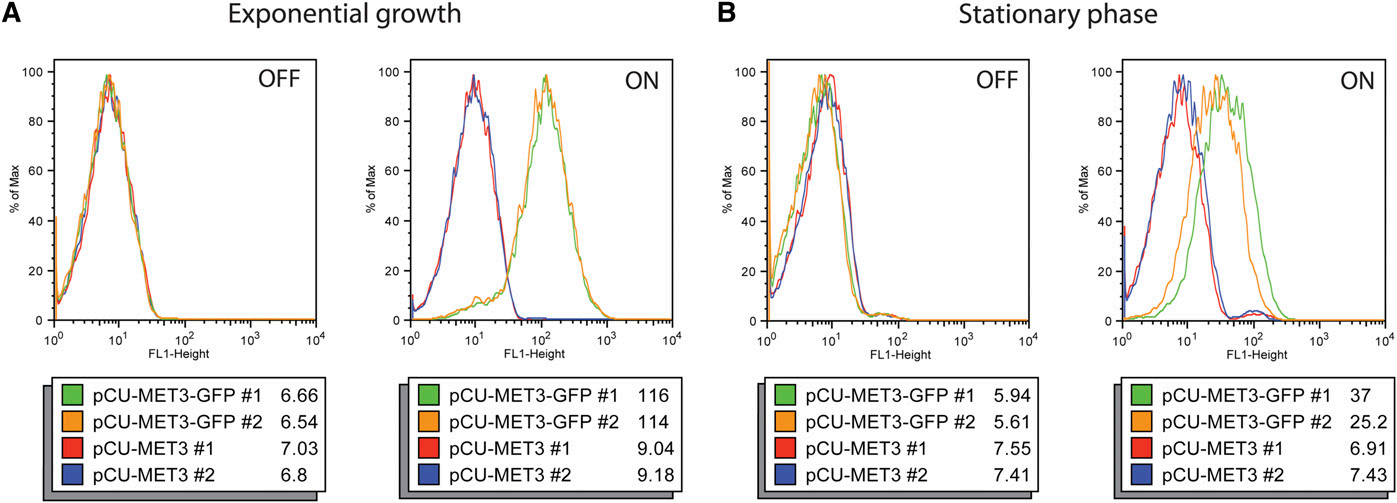
Fluorescence of *C. glabrata* strains carrying pCU-MET3 plasmids. Each panel depicts histograms of fluorescence for two *C. glabrata* strains containing the empty vectors (red and blue lines) and two *C. glabrata* strains containing GFP reporter vectors (green and orange lines). The median fluorescence value for each strain population is shown. “OFF” indicates the strains were grown in media containing 2 mM Met and Cys, which represses the *MET3* promoter. “ON” indicates the strains were grown in media lacking Met and Cys, which induces expression from the *MET3* promoter.

The *MET3* promoter has been used successfully as a regulated promoter in *C. albicans*, and thus the plasmids pCU-MET3 and pCN-MET3 were constructed to allow for control of target gene expression in *C. glabrata* based on the presence or absence of Met and Cys in the media. The control of the *MET3* promoter in *C. glabrata* was monitored by flow cytometry on strains carrying the pCU-MET3 plasmid. In media containing excess Met and Cys (2 mM each), expression is repressed and the fluorescence profiles of pCU-MET3-GFP strains closely match the fluorescence profiles of strains carrying the pCU-MET3 empty vectors (Figure 6, A and B, “OFF”). In media lacking Met and Cys, the expression of GFP was greatly increased in strains carrying the pCU-MET3-GFP plasmids (Figure 6, “ON”). This tight regulation is seen in both log-phase and stationary-phase cultures of strains carrying pCU-MET3-GFP (compare Figure 6A and 6B). Similar results were seen for strains carrying the pCN-MET-GFP plasmids when grown in SED+Met+Cys−Ura or SED−Met−Cys−Ura media (data not shown).

## Discussion

We chose to make a set of “constitutive” plasmids to facilitate cloning and expression of genes in *C. glabrata*. As evidenced by the flow cytometry data monitoring GFP expression in *C. glabrata* strains (Figure 2), the constitutive promoters are of three different strengths, with the rank order being *EGD2*pr<*HHT2*pr<*PDC1*pr.

We also designed versions of our centromeric plasmids with the *MET3* promoter. As in *C. albicans*, this promoter was tightly regulated by the presence or absence of methionine and cysteine in the media (Figure 6). In particular, cells growing exponentially in media supplemented with Met and Cys to repress the *MET3*pr appear to have shut-off all expression; the curves of the pCU-MET3-GFP curves are superimposed on empty vector controls in the far left panel of Figure 6. We note that repression of the *MET3* promoter seems a bit leakier in stationary phase cells. Vectors with the *MET3*pr will allow for tightly controlled expression of a target gene and may be appropriate to use for conditional control of essential genes.

Vectors also were constructed with the phagocytosis-induced promoters *ACO2* and *LYS21*. Expression from these promoters is low when cells are grown in laboratory media (Figure 2, Figure 3, Figure 4). However, 2 hr after engulfment within J774.A1 cultured macrophage cells, expression from the promoters increased. This increase expression is observed with both qRT-PCR and fluorescence microscopy (Figure 4 and Figure 5), and we estimate a 24-fold to 59-fold increase of expression from these promoters after phagocytosis (Figure 4). Increased expression from the *ACO2*pr and *LYS21*pr after phagocytosis likely reflects a response to carbon and amino acid limitation in the phagolysosome (Lorenz *et al.* 2004; Kaur *et al.* 2007). The leaky expression from the *LYS21*pr in stationary phase laboratory media (Figure 3, panel E) may reflect nutrient depletion in a saturated culture. Vectors containing *ACO2*pr or *LYS21*pr may prove useful to express a target gene in yeast specifically within the phagolysosome. Potentially, expression from the ACO2-GFP or LYS21-GFP vectors may serve as indirect indicators of phagocytosis. In the future, it may be possible to develop other plasmids that allow for variable expression within the macrophage or are specific to particular intracellular environments.

The “constitutive” promoters can be used to express proteins at different levels in standard laboratory media in log phase and stationary phase. We noticed that expression from the “constitutive” *PDC1* promoter decreased after phagocytosis by J774A.1 macrophage-like cells (data not shown). Although this is expected based on the transcriptional microarray data comparing phagocytosed and unphagocytosed *C. glabrata* (Kaur *et al.* 2007), it serves to emphasize that expression from the three “constitutive” promoters described in this work should be tested empirically when cells are grown under conditions other than the standard laboratory conditions monitored here.

The plasmids are stably maintained episomally, with no detectable integration into the genome and approximately 5–8% plasmid loss/generation as measured by a plating assay. The pCU vectors maintained at approximately 2 copies per cell; this is comparable with the plasmid copy numbers measured in haploid *S. cerevisiae* cells (Karim *et al.* 2013). The pCN vectors were measured at 0.6–0.8 copies per cell, suggesting that a portion of the yeast cells grown under selection might have lost the pCN plasmid. This may reflect the ability of cells that have lost pCN plasmids to survive and replicate a small number of generations before dying. This is consistent with flow cytometry experiments measuring GFP expression showing that strains carrying pCN-XXX-GFP vectors have a fraction of the population that do not express GFP (data not shown). We conclude that though NAT selection allows overall maintenance of the plasmid, a small fraction of the cells in the culture no longer contain a plasmid, likely resulting from growth of cells that have lost the plasmid for a small number of divisions.

The standardized structure of the plasmids allow for easy modification. A gene of interest cloned into the MCS of one vector can be easily moved to another vector, which could allow researchers to adjust the strength of expression (by choosing a different promoter) or the selectable marker (by choosing the pCU or pCN backbones). For ease, the characteristics of the promoters described in this article are summarized in Table S5. Additionally, the plasmids can be customized by introducing any promoter of choice into either the pCU or the pCN backbone, using a *Sac*I-*Xba*I restriction digest. This flexibility should allow investigators to introduce their favorite gene under control of its native promoter. The set of expression vectors described here can be used in many applications in *C. glabrata* and will expand the cloning tools available to the community.

## Supplementary Material

Supporting Information

Corrigendum
