# Supplementary material for: Expression Plasmids for Use in Candida glabrata
Source: G3 (Bethesda). 2013 Oct 1;3(10):1675–86. doi: 10.1534/g3.113.006908 (PMC3789792; doi:10.1534/g3.113.006908)
Supplement: Supporting Information [file supp_3_10_1675__index.html]

Expression Plasmids for Use in Candida glabrata — Supporting Information 

# Expression Plasmids for Use in *Candida glabrata*

## Supporting Information for Zordan *et al.*, 2013

**Files in this Data Supplement:**

- Supporting Information - Tables S1-S5 (PDF, 505 KB)
- Table S1 - Amino Acid Mixture Recipes (PDF, 425 KB)
- Table S2 - Plasmid copy number (PDF, 310 KB)
- Table S3 - Plasmid loss rates for pCU-PDC1 and pCN-PDC1 (PDF, 331 KB)
- Table S4 - Integration of pCU-PDC1 plasmids into *C. glabrata* genome (PDF, 420 KB)
- Table S5 - Summary of expression from pCU plasmids (PDF, 314 KB)
