# Supplementary material for: Expression Plasmids for Use in Candida glabrata
Source: G3 (Bethesda). 2013 Oct 1;3(10):1675–86. doi: 10.1534/g3.113.006908 (PMC3789792; doi:10.1534/g3.113.006908)
Supplement: Supporting Information [file supp_g3.113.006908_006908SI.pdf]

G3 · Genes | Genomes | Genetics

Expression plasmids for use in *Candida glabrata*

Rebecca Zordan[*], Yuxia Ren[*], Shih-Jung Pan[*], Giuseppe Rotondo[*,1], Alejandro De Las Peñas[*,2], Joseph Iluore[*], Brendan Cormack[*, 3]

[*]Department of Molecular Biology and Genetics, Johns Hopkins University School of Medicine, Baltimore, Maryland, 21205

[1] Current address: Dipartimento di Bioscienze, Università degli Studi di Milano, Milano, Italy, 20133

[2] Current address: IPICYT, División de Biología Molecular, Instituto Potosino de Investigación Científica y Tecnológica, San Luis Potosí, México

[3]Corresponding author: 725 N Wolfe St. Hunterian 617, Baltimore, MD 21205. Email: bcormack@jhmi.edu

DOI: 10.1534/g3.113.006908

Table S1 Amino Acid Mixture Recipes

Component (mg/L)	SC*	-Met -Cys -Ura	+Met +Cys -Ura (2mM Met/Cys)
Adenine	21	21	21
L-Alanine	85.6	85.6	85.6
L-Arginine	85.6	85.6	85.6
L-Asparagine	85.6	85.6	85.6
L-Aspartic Acid	85.6	85.6	85.6
L-Cysteine	85.6	0	242.32
Glutamine	85.6	85.6	85.6
L-Glutamic Acid	85.6	85.6	85.6
Glycine	85.6	85.6	85.6
L-Histidine	85.6	85.6	85.6
Myo-Inositol	85.6	85.6	85.6
L-Isoleucine	85.6	85.6	85.6
L-Leucine	173.4	173.4	173.4
L-Lysine	85.6	85.6	85.6
L-Methionine	85.6	0	298.42
Para-AminoBenzoic Acid (PABA)	8.6	8.6	8.6
L-Phenylalenine	85.6	85.6	85.6
L-Proline	85.6	85.6	85.6
L-Serine	85.6	85.6	85.6
L-Threonine	85.6	85.6	85.6
L-Tryptophan	85.6	85.6	85.6
L-Tyrosine	85.6	85.6	85.6
Uracil	85.6	**0**	**0**
L-Valine	85.6	85.6	85.6
TOTAL (mg/L media)	**2000.6**	**1743.8**	**2284.54**
Use g mix/ L media	2	1.74	2.28

* SC = Synthetic Complete Supplement Mixture (amino acids)

Table S2 Plasmid copy number

Plasmid	Average copy number
pCU-EGD2	2.51 ± 0.22
pCU-ACO2	2.11 ± 0.18
pCN-EGD2	0.58 ± 0.015
pCN-ACO2	0.78 ± 0.14

Total DNA was isolated from *C. glabrata* strains carrying pCU-EGD2, pCU-ACO2, pCN-EGD2, or pCN-ACO2 (2 strains each). Quantitative PCR was used to measure relative amounts of Ap^R and *TUB1* DNA in each sample to monitor plasmid and genomic DNA, respectively. Quantitative PCR was performed in triplicate for each sample and primer set. Average Ap^R and *TUB1* starting quantities were calculated for each strain, and a ratio of Ap^R/*TUB1* was used to calculate the plasmid copy number per cell in a given strain. The average and standard deviation of Ap^R/*TUB1* ratios between replicate strains are shown above, to represent the average copy number for each plasmid.

Table S3 Plasmid loss rates for pCU-PDC1 and pCN-PDC1

t (hrs)	Strain	Plasmid	# colonies on selective media after replica plating		Total colonies	Fraction of population with plasmid	t10/t0 (relative fraction of population with plasmid)	# gener-ations in 10 hrs	% plasmid loss/ generation
			Growth	No growth					
0	BG3320	pCU-PDC1	352	76	428.0	0.822	0.67	9.27	4.3%
0	BG3321	pCU-PDC1	383	64	447.0	0.857	0.63	9.26	4.9%
0	BG3332	pCN-PDC1	329	136	465.0	0.708	0.46	9.72	7.6%
0	BG3333	pCN-PDC1	284	125	409.0	0.694	0.58	9.74	5.4%
10	BG3320	pCU-PDC1	204	168	372.0	0.548			
10	BG3321	pCU-PDC1	152	130	282.0	0.539			
10	BG3332	pCN-PDC1	127	260	387.0	0.328			
10	BG3333	pCN-PDC1	147	215	362.0	0.406			

C. glabrata strains carrying pCU-PDC1 or pCN-PDC1 were grown in selective media until t=0, at which point the cultures were washed and resuspended in YPD (non-selective) media. After 5 hours growth at 30°C, OD_{600} readings were taken and the cultures were diluted into fresh media at OD_{600}=0.02 and incubated at 30°C. At 10 hours growth, OD_{600} readings were taken and cells were plated onto three YPD plates and grown for 1-2d at 30°C. The YPD plates were replica-plated onto appropriate selective media; the selective plates were grown for 2d at 30°C. Colonies which totally failed to grow on selective media are scored as “No growth” in the table above. The “Fraction of the population with the plasmid” was calculated as “Growth”/”Total colonies” ratio. The OD_{600} readings were used to calculate the number of generations that had elapsed between t=0 and t=10 hours. The “percent plasmid loss per generation” was calculated for each strain as described in the Material and Methods.

Table S4 Integration of pCU-PDC1 plasmids into *C. glabrata* genome

Strain	Plasmid	5-FOA^S colonies	5-FOA^R colonies	Total colonies	Rate of integration into genome
BG3320	pCU-PDC1	0	1115	1115	< 1/1115 = <0.09%
BG3321	pCU-PDC1	0	1226	1226	< 1/1226 = <0.08%
TOTAL	pCU-PDC1	**0**	**2341**	**2341**	**<1/2341 = <0.04%**

Saturated cultures of *C. glabrata* strains carrying pCU-PDC1 were plated and grown SD-Ura plates, then replica-plated onto 5-FOA plates. Comparison of colony growth between the SD-Ura and 5-FOA plates led to the designation of colonies as 5-FOA^S or 5-FOA^R. Sensitivity to 5-FOA would indicate the *URA3* marker from pCU-PDC1 could not be lost and suggests it had been integrated into the genome.

Table S5 Summary of expression from pCU plasmids

<table>
<tr><th rowspan="2">Promoter</th><th rowspan="2">Description</th><th colspan="4">Expression
(Relative fluorescence units)</th><th rowspan="2">Fold Induction after phago-cytosis</th></tr>
<tr><td colspan="2">Exponential phase</td><td colspan="2">Stationary phase</td></tr>
<tr><td>EGD2</td><td>Constitutive</td><td colspan="2">25.4</td><td colspan="2">11.0</td><td>nd</td></tr>
<tr><td>HHT2</td><td>Constitutive</td><td colspan="2">85.1</td><td colspan="2">25.5</td><td>nd</td></tr>
<tr><td>PDC1</td><td>Constitutive</td><td colspan="2">197</td><td colspan="2">76.7</td><td>nd</td></tr>
<tr><td>ACO2</td><td>Macrophage-induced</td><td colspan="2">3.74</td><td colspan="2">4.54</td><td>24x</td></tr>
<tr><td>LYS21</td><td>Macrophage-induced</td><td colspan="2">30.6</td><td colspan="2">42.0</td><td>30-59x</td></tr>
<tr><td rowspan="2">MET3</td><td rowspan="2">Nutritionally-regulated</td><td>Repressed</td><td>Induced</td><td>Repressed</td><td>Induced</td><td rowspan="2">nd</td></tr>
<tr><td>0*</td><td>106</td><td>0*</td><td>23.8</td></tr>
</table>

This table includes data from Figures 2, 3, 4, and 6 and summarizes characteristics of different promoters. The "Expression" in exponential and stationary phase is the median GFP signal (average of two independent strains carrying pCU-XXX-GFP plasmids) less the median signal from matched non-fluorescent strains, as measured by flow cytometry. "Fold induction after phagocytosis" is the ratio of normalized GFP transcript levels in cells following phagocytosis and cells grown in TC media (measured by RTqPCR). * Values calculated were <0. nd = not determined.
