# Supplementary material for: Expression Plasmids for Use in Candida glabrata
Source: G3 (Bethesda). 2013 Oct 1;3(10):1675–86. doi: 10.1534/g3.113.006908 (PMC3789792; doi:10.1534/g3.113.006908)
Supplement: Supporting Information [file supp_g3.113.006908_TableS1.pdf]

Table S1 Amino Acid Mixture Recipes

Component (mg/L)	SC*	-Met -Cys -Ura	+Met +Cys -Ura (2mM Met/Cys)
Adenine	21	21	21
L-Alanine	85.6	85.6	85.6
L-Arginine	85.6	85.6	85.6
L-Asparagine	85.6	85.6	85.6
L-Aspartic Acid	85.6	85.6	85.6
L-Cysteine	85.6	0	242.32
Glutamine	85.6	85.6	85.6
L-Glutamic Acid	85.6	85.6	85.6
Glycine	85.6	85.6	85.6
L-Histidine	85.6	85.6	85.6
Myo-Inositol	85.6	85.6	85.6
L-Isoleucine	85.6	85.6	85.6
L-Leucine	173.4	173.4	173.4
L-Lysine	85.6	85.6	85.6
L-Methionine	85.6	0	298.42
Para-AminoBenzoic Acid (PABA)	8.6	8.6	8.6
L-Phenylalenine	85.6	85.6	85.6
L-Proline	85.6	85.6	85.6
L-Serine	85.6	85.6	85.6
L-Threonine	85.6	85.6	85.6
L-Tryptophan	85.6	85.6	85.6
L-Tyrosine	85.6	85.6	85.6
Uracil	85.6	**0**	**0**
L-Valine	85.6	85.6	85.6
TOTAL (mg/L media)	**2000.6**	**1743.8**	**2284.54**
Use g mix/ L media	2	1.74	2.28

* SC = Synthetic Complete Supplement Mixture (amino acids)
