# Supplementary material for: Expression Plasmids for Use in Candida glabrata
Source: G3 (Bethesda). 2013 Oct 1;3(10):1675–86. doi: 10.1534/g3.113.006908 (PMC3789792; doi:10.1534/g3.113.006908)
Supplement: Supporting Information [file supp_g3.113.006908_TableS2.pdf]

Table S2   Plasmid copy number

Plasmid	Average copy number
pCU-EGD2	2.51 ± 0.22
pCU-ACO2	2.11 ± 0.18
pCN-EGD2	0.58 ± 0.015
pCN-ACO2	0.78 ± 0.14

Total DNA was isolated from *C. glabrata* strains carrying pCU-EGD2, pCU-ACO2, pCN-EGD2, or pCN-ACO2 (2 strains each). Quantitative PCR was used to measure relative amounts of Ap^R and *TUB1* DNA in each sample to monitor plasmid and genomic DNA, respectively. Quantitative PCR was performed in triplicate for each sample and primer set. Average Ap^R and *TUB1* starting quantities were calculated for each strain, and a ratio of Ap^R/*TUB1* was used to calculate the plasmid copy number per cell in a given strain. The average and standard deviation of Ap^R/*TUB1* ratios between replicate strains are shown above, to represent the average copy number for each plasmid.
