# Supplementary material for: Expression Plasmids for Use in Candida glabrata
Source: G3 (Bethesda). 2013 Oct 1;3(10):1675–86. doi: 10.1534/g3.113.006908 (PMC3789792; doi:10.1534/g3.113.006908)
Supplement: Supporting Information [file supp_g3.113.006908_TableS3.pdf]

Table S3 Plasmid loss rates for pCU-PDC1 and pCN-PDC1

t (hrs)	Strain	Plasmid	# colonies on selective media after replica plating		Total colonies	Fraction of population with plasmid	t10/t0 (relative fraction of population with plasmid)	# gener-ations in 10 hrs	% plasmid loss/ generation
			Growth	No growth					
0	BG3320	pCU-PDC1	352	76	428.0	0.822	0.67	9.27	4.3%
0	BG3321	pCU-PDC1	383	64	447.0	0.857	0.63	9.26	4.9%
0	BG3332	pCN-PDC1	329	136	465.0	0.708	0.46	9.72	7.6%
0	BG3333	pCN-PDC1	284	125	409.0	0.694	0.58	9.74	5.4%
10	BG3320	pCU-PDC1	204	168	372.0	0.548			
10	BG3321	pCU-PDC1	152	130	282.0	0.539			
10	BG3332	pCN-PDC1	127	260	387.0	0.328			
10	BG3333	pCN-PDC1	147	215	362.0	0.406			

C. glabrata strains carrying pCU-PDC1 or pCN-PDC1 were grown in selective media until t=0, at which point the cultures were washed and resuspended in YPD (non-selective) media. After 5 hours growth at 30°C, OD_{600} readings were taken and the cultures were diluted into fresh media at OD_{600}=0.02 and incubated at 30°C. At 10 hours growth, OD_{600} readings were taken and cells were plated onto three YPD plates and grown for 1-2d at 30°C. The YPD plates were replica-plated onto appropriate selective media; the selective plates were grown for 2d at 30°C. Colonies which totally failed to grow on selective media are scored as "No growth" in the table above. The "Fraction of the population with the plasmid" was calculated as "Growth"/"Total colonies" ratio. The OD_{600} readings were used to calculate the number of generations that had elapsed between t=0 and t=10 hours. The "percent plasmid loss per generation" was calculated for each strain as described in the Material and Methods.
