# Supplementary material for: Expression Plasmids for Use in Candida glabrata
Source: G3 (Bethesda). 2013 Oct 1;3(10):1675–86. doi: 10.1534/g3.113.006908 (PMC3789792; doi:10.1534/g3.113.006908)
Supplement: Supporting Information [file supp_g3.113.006908_TableS4.pdf]

Table S4 Integration of pCU-PDC1 plasmids into *C. glabrata* genome

Strain	Plasmid	5-FOA^S colonies	5-FOA^R colonies	Total colonies	Rate of integration into genome
BG3320	pCU-PDC1	0	1115	1115	< 1/1115 = <0.09%
BG3321	pCU-PDC1	0	1226	1226	< 1/1226 = <0.08%
TOTAL	pCU-PDC1	**0**	**2341**	**2341**	**<1/2341 = <0.04%**

Saturated cultures of *C. glabrata* strains carrying pCU-PDC1 were plated and grown SD-Ura plates, then replica-plated onto 5-FOA plates. Comparison of colony growth between the SD-Ura and 5-FOA plates led to the designation of colonies as 5-FOA^S or 5-FOA^R. Sensitivity to 5-FOA would indicate the *URA3* marker from pCU-PDC1 could not be lost and suggests it had been integrated into the genome.
