# Supplementary material for: Expression Plasmids for Use in Candida glabrata
Source: G3 (Bethesda). 2013 Oct 1;3(10):1675–86. doi: 10.1534/g3.113.006908 (PMC3789792; doi:10.1534/g3.113.006908)
Supplement: Supporting Information [file supp_g3.113.006908_TableS5.pdf]

Table S5   Summary of expression from pCU plasmids

<table>
<tr><th rowspan="2">Promoter</th><th rowspan="2">Description</th><th colspan="4">Expression
(Relative fluorescence units)</th><th rowspan="2">Fold Induction after phago-cytosis</th></tr>
<tr><td colspan="2">Exponential phase</td><td colspan="2">Stationary phase</td></tr>
<tr><td>EGD2</td><td>Constitutive</td><td colspan="2">25.4</td><td colspan="2">11.0</td><td>nd</td></tr>
<tr><td>HHT2</td><td>Constitutive</td><td colspan="2">85.1</td><td colspan="2">25.5</td><td>nd</td></tr>
<tr><td>PDC1</td><td>Constitutive</td><td colspan="2">197</td><td colspan="2">76.7</td><td>nd</td></tr>
<tr><td>ACO2</td><td>Macrophage-induced</td><td colspan="2">3.74</td><td colspan="2">4.54</td><td>24x</td></tr>
<tr><td>LYS21</td><td>Macrophage-induced</td><td colspan="2">30.6</td><td colspan="2">42.0</td><td>30-59x</td></tr>
<tr><td rowspan="2">MET3</td><td rowspan="2">Nutritionally-regulated</td><td>Repressed</td><td>Induced</td><td>Repressed</td><td>Induced</td><td rowspan="2">nd</td></tr>
<tr><td>0*</td><td>106</td><td>0*</td><td>23.8</td></tr>
</table>

This table includes data from Figures 2, 3, 4, and 6 and summarizes characteristics of different promoters. The “Expression” in exponential and stationary phase is the median GFP signal (average of two independent strains carrying pCU-XXX-GFP plasmids) less the median signal from matched non-fluorescent strains, as measured by flow cytometry. “Fold induction after phagocytosis” is the ratio of normalized GFP transcript levels in cells following phagocytosis and cells grown in TC media (measured by RTqPCR). * Values calculated were <0. nd = not determined.
