# Supplementary material for: Expression Plasmids for Use in Candida glabrata
Source: G3 (Bethesda). 2013 Oct 1;3(10):1675–86. doi: 10.1534/g3.113.006908 (PMC3789792; doi:10.1534/g3.113.006908)
Supplement: Corrigendum [file supp_3_10_1675_v2_index.html]

Corrigendum 

# Expression Plasmids for Use in *Candida glabrata*

## Corrigendum for Zordan *et al.*, G3: Genes|Genomes|Genetics 3 (10) 1675-1686.

**Files in this Data Supplement:**

- Corrigendum - Corrigendum for Zordan *et al.*, G3: Genes|Genomes|Genetics 3 (10) 1675-1686.
