# Supplementary material for: Expression Plasmids for Use in Candida glabrata
Source: G3 (Bethesda). 2013 Oct 1;3(10):1675–86. doi: 10.1534/g3.113.006908 (PMC3789792; doi:10.1534/g3.113.006908)
Supplement: Corrigendum [file supp_g3.113.006908_Corrigendum_Zordan.pdf]

Corrigendum for Zordan *et al.*, *G3* 3 (10) 1675-1686.

G3, Vol 3, 1675-1686, October 2013, Copyright © 2013 Genetics Society of America.

CORRIGENDUM

In the article by R. E. Zordan, Y. Ren, S.-J. Pan, G. Rotondo, A. De Las Peñas, *et al.* (G3 3: 1675-1686) entitled "Expression Patterns for Use in *Candida glabrata*", a set of nucleotide coordinates in Plasmid Construction section of Materials and Methods have been modified.

In the second paragraph of the Plasmid construction section, the third sentence read:

The ARS sequence was functionally isolated from *C. glabrata* strain BG2 and corresponds to nucleotides 954976-955403 of chromosome F in the published *C. glabrata* CBS138 sequence.

The corrected sentence now reads:

The ARS sequence was functionally isolated from *C. glabrata* strain BG2 and corresponds to nucleotides 286060-286210 of chromosome F in the published *C. glabrata* CBS138 sequence.
